# Innovative Preservation Technology for the Fresh Fruit and Vegetables

**DOI:** 10.3390/foods10040719

**Published:** 2021-03-29

**Authors:** Bernardo Pace, Maria Cefola

**Affiliations:** Institute of Sciences of Food Production, National Research Council of Italy (CNR), c/o CS-DAT, Via M. Protano, 71121 Foggia, Italy; maria.cefola@ispa.cnr.it

**Keywords:** active cardboard box, antimicrobial compounds, biocontrol, nanoparticles coating, oxalic acid, plasma-activated water, preservatives, respiration rate, shelf life, TiO_2_ photocatalytic

## Abstract

The preservation of the freshness of fruits and vegetables until their consumption is the aim of many research activities. Quality losses of fresh fruit and vegetables during cold chain are frequently attributable to an inappropriate use of postharvest technologies. Moreover, especially when fresh produce is transported to distant markets, it is necessary to adopt proper postharvest preservation technologies in order to preserve the initial quality and limit microbial decay. Nowadays, for each step of supply chain (packing house, cold storage rooms, precooling center, refrigerate transport and distribution), are available innovative preservation technologies that, alone or in combination, could improve the fresh products in order to maintain the principal quality and nutritional characteristics. The issue groups five original studies and two comprehensive reviews within the topic of preservation technologies related to innovative packaging and postharvest operation and treatments, highlighting their effect on quality keeping.

Fresh fruits and vegetables are perishable food, which undergo quality losses during cold chain if suitable preservation technologies are not used. Among these, packaging has an important role. Recently, active packaging was successful applied to improve the shelf life of fresh table grape [1] and blueberries [2]. Furthermore, active packaging, using red thyme oil (*Thymus vulgaris* L.), could be employed by the citrus industry to extend the shelf-life of oranges for fresh market use and juice processing [3].

Moreover, postharvest processes and technologies may preserve the quality and limit microbial decay in fresh fruits and vegetables. In this context, Pinto et al. [4] showed that the exposure to gaseous ozone before packaging followed by storage under modified atmosphere packaging (MAP) could be a useful technological approach to extend the postharvest storage of small berry fruit. Similarly, the combined effect of cold storage and oxalic acid treatment resulted in a valid and sustainable solution to preserve the visual quality of green and purple asparagus spears [5].

In the Special Issue on “Innovative Preservation Technology for the Fresh Fruit and Vegetables”, three main research topics were covered: (i) innovative packaging; (ii) postharvest processes and technology affecting the product quality; (iii) postharvest technology to limit microbial decay. Within the first topic, three research articles were published.

In the first article [6], the use of cardboard box activated with essential oils in order to enhance the shelf life of fresh mandarins (*Citrus reticulata* Murcott Seedless) was described. The authors studied the effect of different forms of paperboard packages sprayed in internal surfaces with the lacquer containing essential oils (EOs) encapsulated by cyclodextrins inclusion complex in order to simulate the transport boxes of 10 kg of mandarins fruits until three weeks at 8 °C. This results in reduced microbial growth (together with decay incidence) and weight losses, while maintaining the physicochemical quality (soluble solids, titratable acidity, firmness and color) with an increase of one week in storage life. The controlled release of EOs from the box extended the shelf life of mandarins during either a long cold transportation simulation or a commercialization period at non-recommended room temperature.

In the second paper, Pace et al. [7] studied the proper oxalic acid (OA) concentration and the suitable packaging material able to maintain an adequate low O_2_ concentration inside fresh-cut iceberg lettuce (*Lactuca sativa* L.) bags closed in MAP. The results showed a significant effect of 5 mM OA on respiration rate delay. In addition, polypropylene/polyamide (PP/PA) was selected as the most suitable packaging material to be used in low O_2_ MAP. Combining OA dipping with low O_2_ MAP using PP/PA as material resulted in the ability to reduce leaf edge browning, respiration rate, weight loss and electrolyte leakage, preserving the visual quality of fresh-cut lettuce until 8 days at 8 °C.

The last article, within the first topic, aimed to identify the impact of 15% Kelulut honey (KH) nanoparticles (Nps) coating solution on papaya’s respiration rate, antioxidant activity and total phenol content and to investigate the respiration rate kinetic model of nano-coated papaya (*Carica papaya* L.) using the Peleg model in order to describe the function of gas composition and storage day [8]. The results showed that KH Nps coating can be used as a conserving material, extending the shelf life by inhibiting the respiration rate and C_2_H_4_ production, while maintaining the antioxidant activity and total phenol content, in papaya.

The research topic regarding postharvest processes and technology affecting the product quality was also covered with two research articles published in this Special Issue.

The effect of cutting styles (slice, pie and shred) on the quality characteristics and antioxidant activity of purple and yellow flesh sweet potato cultivars during 6 days of storage at 4 °C was investigated by Dovene et al. [9]. The finding of this study revealed that pie-cut processing has potential in improving the quality and increasing the antioxidant activity of fresh-cut purple and yellow flesh sweet potato (*Ipomoea batatas* L.) cultivars, while shredding accelerated the quality deterioration of both sweet potato cultivars. Jia et al. [10] proposed a precise temperature control cold storage with low-temperature fluctuation (LFT) combined with an ozone (O_3_) generator and a titanium dioxide (TiO_2_) photocatalytic reactor to the cold storage of peach (*Prunus persica* L. Batsch). The results showed that LFT significantly reduced the chilling injury of peach fruit during storage. Moreover, its combination with the TiO_2_ photocatalytic system significantly improved the postharvest storage quality of the fruit. This treatment maintained higher titratable acidity, total soluble solids, better firmness, color, microstructure and lower decay rate, polyphenol oxidase activities, total phenol accumulation, respiratory intensity, ethylene production and malondialdehyde content during 60 d of storage.

Finally, the last research topic was the object of two reviews. In the first one, the recent physical, chemical and the biological approaches conceived to control the development of gray mold *Botrytis cinerea* in table grapes was discussed by De Simone et al. [11]. Since the global consumption of table grape (*Vitis vinifera* L.) has increased in the last 20 years by over 70% [12], the researchers studied solutions to control *Botrytis cinerea,* which represent the major cause of table grapes losses occurring in pre and postharvest. Among physical methods, dipping in hot water, electrolyzed oxidizing water or different gas compositions using controlled or modified atmosphere packaging are discussed. Regarding the chemical methods, different treatments (wound inoculation, spraying, dipping or fumigation) were reported to control *Botrytis cinerea*. In regard to the bio-based applications, several protective cultures and compounds of biological origin, were assessed for their possible use as biological control agents against gray mold decay. Many biological compounds were tested for the biocontrol of table grape spoilages and these compounds include vegetal extracts, essential oils or edible coating. The authors highlight that each treatment has peculiar benefits and limitations that affect the concrete applications and the future perspectives. As previously applied with success in other fields, an integrated management program that contemplate the combinations of two or more different solutions, could be useful to minimize post-harvest losses caused by undesired fungal development on table grape.

The second review elaborated the properties of plasma-activated water (PAW), the effect of various treatment parameters on its efficiency in bacterial inactivation and its usage as a standalone technology, as well as a hurdle approach with mild thermal treatments [13]. A section highlighting different models that can be employed to generate PAW alongside a direct comparison of the PAW characteristics on the inactivation potential and the existing research gaps are also included. The mechanism of action of PAW on the bacterial cells and any reported effects on the sensory qualities and shelf life of food has been evaluated. It was concluded that PAW offers a significant potential as a non-chemical and non-thermal intervention for bacterial inactivation, especially on food. However, the applicability and usage of PAW depend on the effect of environmental and bacterial strain-based conditions and cost-effectiveness.

In conclusion, the research papers proposed, carried out within the European or National Project or promoted by private enterprise, reported innovative results improving the knowledge in the topic of “Innovative Preservation Technology for the Fresh Fruit and Vegetables”. We expect the results presented in this Special Issue to be a stimulus for other future research in this field.

## Data Availability

Not applicable.
